# Family roles and limitations as mediators in medical communication for cancer patients: A qualitative study

**DOI:** 10.1371/journal.pone.0333669

**Published:** 2025-10-14

**Authors:** Yeori Park, Jiyeon Kang, Yeonju Kim, Young Su Park, Sang-Ho Yoo

**Affiliations:** 1 Institute of Humanities, University of Seoul, Seoul, Republic of Korea; 2 Department of Medical Humanities and Education, School of Medicine, Kyungpook National University, Daegu, Republic of Korea; 3 Department of Anthropology, Seoul National University, Seoul, Republic of Korea; 4 Department of the History of Medicine and Medical Humanities, Seoul National University, Seoul, Republic of Korea; 5 Department of Medical Humanities and Ethics, Hanyang University College of Medicine, Seoul, Republic of Korea; Touro University California College of Pharmacy, UNITED STATES OF AMERICA

## Abstract

This qualitative study explored the role and limitations of families as mediators of medical communication among cancer patients in South Korea. Semi-structured interviews were conducted with 46 participants including patients, family members, doctors, and nurses. This study examined how families alleviate communication burdens and navigate power dynamics between patients and physicians. The findings revealed that families gather, interpret, and translate medical information, often acting as intermediaries, owing to limited consultation times and complex medical terminology. They provide emotional, financial, and integrated care; influence treatment decisions; and ensure compliance. However, the study also identified limitations, such as disagreements among family members, information concealment, and the potential undermining of patient autonomy. This study underscores the need for nuanced approaches to family involvement that prioritize patient-centered care and respect individual preferences.

## Introduction

Cancer is the leading cause of death among Koreans. According to the 2021 statistics, lung and bronchial cancers are the leading cause of death in men and the sixth leading cause of death in women [1]. According to nationwide cancer incidence statistics from 1999 to 2022, the number of cancer patients in South Korea has reached approximately 2.5 million [2], with projections indicating approximately 230,000 new cases by 2024 [3]. This study analyzed how families address the burden of medical communication faced by patients with cancer during treatment. Families play a crucial role by intervening at every stage of medical communication from before diagnosis to after treatment, thereby alleviating potential issues.

Cancer often requires years of treatment and follow-up even when surgery is possible. Effective communication is critical in cancer care because of the complexity and emotional impact of the disease. Even with precise treatment, poor communication may result in irregular follow-up, making it challenging to achieve successful treatment. From healthcare providers’ perspective, smooth communication is essential for understanding disease progression and patient management, which also affect treatment outcomes [4]. Positive and effective communication with physicians has been shown to alleviate stress and depression in patients with cancer [5], improve their health status [6], and motivate them to actively engage in treatment [4]. This, in turn, enhances treatment adherence.

However, several barriers hinder effective communication among doctors, patients with cancer, and their families in East Asian medical settings. Previous studies identified the following factors impeding doctor–patient communication: insufficient consultation time because of fast-paced appointments [7], lack of necessary information [8], limitations in discussions caused by information asymmetry [8], and inability to voice opinions due to doctors’ authoritative attitudes [9]. Research indicates that in East Asian cultures, the paternalistic authority held by doctors as experts might create challenging clinical environments for patients [10].

However, patients may not passively accept these challenges. They leverage various forms of social capital to bridge the information gap. For instance, they may bring family members or friends to clinical consultations to help interpret complex information [11], gather medical information through online platforms before consultations [12,13], communicate primarily with nurses [14], or seek advice from healthcare professionals on social networks. In this process, family members often serve as primary and immediate mediators facilitating communication between patients and doctors.

In addition to the patients’ strategic actions, families often act as key agents in the medical decisions of cancer patients in South Korea [15,16]. According to Lee et al. [17], in Korea, over 80% of patients with cancer and more than 70% of their families believe that they should be fully informed about the overall treatment process. In cases of disagreement over treatment decisions, 14% of patients and 22% of family members prioritized family opinions over patient opinions. This indicates that family opinions may override patient consent, highlighting the influence of family members on medical communication in South Korea [18].

This study captures the specific ways in which families alleviate the communication burdens faced by patients and serve as mediators between doctors and patients [19]. Much of the health communication conducted by laypeople outside of professional settings is co-produced through the labor of care [20]. This study examined how families alleviate the burdens that arise in communication within South Korea’s authoritative and rapidly changing healthcare environment, providing support for communication that extends beyond cancer treatment and encompasses the lives of patients as individuals.

## Materials and methods

### Design

Data were collected through semi-structured interviews. The interviews covered the experiences of patients with cancer and their caregivers regarding medical treatments and decision-making processes.

### Recruitment

The interview subjects were chosen from among patients with cancer who had received treatment at a university hospital and their families, regardless of the cancer type. The study participants were recruited between May 29, 2024 and December 31, 2024. With permission from the two university hospitals, recruitment announcements were posted on bulletin boards to attract participants. The participants were recruited using snowball sampling. Of the 46 interview participants, there were 15 patients, 15 family members, ten medical doctors, and six nurses (Table 1). Among them, all individuals aged 60 or older were either patients or family members. Patients with stage I cancer underwent surgery only, whereas the remaining patients received both chemotherapy and radiation therapy. Participants 010, 014, and 043 were enrolled in a clinical trial.

**Table 1:** Demographic characteristics of the research subjects.

Characteristics		N = 46
Sex		%
Male	12	26.09
Female	34	73.91
Category		
Patient	15	32.61
Family	15	32.61
Doctor	10	21.74
Nurse	6	13.04
Age (years)		
19–29	6	13.04
30–39	15	32.61
40–49	13	28.26
50–59	6	13.04
60–69	5	10.87
70–	1	2.17
Cancer	N = 15 (Patient)	
Breast cancer	8	53.33
Lung cancer	1	6.67
Thyroid cancer	2	13.33
Duodenal cancer	1	6.67
Renal cancer	1	6.67
Colorectal cancer	1	6.67
Leukemia	1	6.67
Cancer stage		
Stage 1	6	40.00
Stage 2	1	6.67
Stage 3	4	26.67
Stage 4	3	20.00
Unidentified	1	6.67

Because all research participants were independently recruited within each category, the patients, family members, and physicians were not directly related to each other, except for one case in which a patient introduced their own family member as an interviewee through snowball sampling. Participants were recruited until an adequate sample size was achieved and no new themes emerged from the interviews, at which point recruitment was discontinued.

Once the interview participants were contacted, a written informed consent form was provided prior to the interview. The researcher reviewed the content with the participants, explained the details, and ensured that they fully understood the information before obtaining their signatures on a consent form. After obtaining written consent, interviews were conducted. All interviews with patients and nurses were conducted face-to-face, whereas some interviews with physicians were conducted via video conferencing. The interviews lasted between 60 and 120 min each. Interviews were conducted in reserved spaces to ensure privacy. The researchers participated in all the key interviews and were assigned specific interviewees to conduct. All researchers were experts with prior experience in qualitative healthcare research. Participants received a modest honorarium as compensation for their participation. The interviews were audio recorded using a digital recorder. The initial transcription was completed using an AI-based transcription application, and the research team reviewed and verified the entire transcript for accuracy. The interviews were recorded with the participants’ consent. All personal identifying information was removed from the interview transcripts and stored in an encrypted format.

### Data collection

Because the interviews were semi-structured, broad questions were asked first. After the participants completed the questionnaire, they were asked additional questions based on their responses. An analysis of the interviews revealed that when broadly based questions were asked about the diagnosis and treatment processes without specifying the family members, the family emerged as a key participant. Consequently, follow-up questions were asked to explore the family’s influence on decision-making and their experiences in this context. The following questions were asked of the patients and their family members:

1. Can you explain what happened to you before you went to the hospital and why you initially sought out a doctor?
  Who did you share this story with?
  Who accompanied you to the medical appointment?
2. Can you tell me about the diagnostic process?
3. What experiences did you have in the treatment process?
4. Can you explain how the discussion regarding treatment options was conducted?
  Did your family or friends participate in making the treatment decisions? If so, could you please describe their roles?
  Do you believe you or your family should be more involved in the decision-making process?

The following questions were asked to medical practitioners:

1. Can you tell us about the diagnostic process?
  What is your relationship with the patient in your care?
  Did you feel that there was sufficient time for consultation?
  Did you experience any difficulty communicating with the patient? If so, what kinds of difficulties did you encounter?
2. Could you tell us about the decision-making process for treatment?
  What treatment options are currently available?
  What factors influence the treatment decisions?
  What is your role in decision-making?
3. Did the patient, family members, or friends participate in making the treatment decisions? If so, can you describe the roles that you played?
4. Who do you think should make the final treatment decision?
  Who made the final decisions?
  Do you think that the final decision is appropriate for the patient’s situation?

### Analysis

The data were coded using MAXQDA [21], a qualitative research analysis program. Coding was conducted based on grounded theory, which follows an inductive approach by coding the data without pre-establishing a hypothesis, and using the findings as a basis for analysis [22]. This method was suitable for this study, as it could derive common challenges and familiar support based on various experiences of medical communication involving cancer patients. In the open coding stage, the researcher (YP) initially coded the interviews. The researcher selected common major themes based on a semi-structured questionnaire and created approximate subcategories within these themes. Subsequently, the contents of the open coding were finalized at a meeting involving the entire research team. In the axial coding phase, the team members JYK, YJK, YRP, YSP, and SHY reviewed the results of the open coding and classified the interview content into subcategories. The appropriateness of the classified items was subsequently evaluated during team meetings. Finally, in the selective coding phase, the entire research team finalized the core categories of the study, which encompassed all categories, and reviewed the appropriateness of the detailed categories. The codebook was reviewed through a process in which all the researchers individually confirmed it repeatedly and gathered opinions at overall meetings. The codebook was completed after several supervision sessions with external advisory members and the corresponding authors (YSP and SHY).

### Ethics

This study was approved by the Institutional Review Boards of Seoul National University Hospital and Seoul National University Bundang Hospital (no. 2401-161-1505). The ethics committee reviewed and approved the recruitment methods and ethical issues related to the selection of research participants and methods.

## Results

### The role of family in the medical communication process

The findings indicated that the family plays a crucial role in the communication process of cancer patients in South Korea. Given that cancer treatment often involves prolonged and physically demanding interventions, the family’s role is more significant than that for other diseases. Families serve as active agents in addressing and resolving communication issues in healthcare, intervening across the entire spectrum of medical interactions, from the initial visit to a physician to the management of aftereffects and caregiving after treatment.

The study revealed that due to certain limitations in Korean medical culture, families often compensate for the various challenges encountered by patients in the decision-making process (Table 2). For instance, because of the limited consultation time and the use of complex medical terminology, families frequently provide medical information to patients outside clinical settings. Additionally, because physicians do not always provide sufficient emotional support, families may fulfill this role and help patients cope with their condition.

**Table 2:** Themes that emerged from coding.

Main Theme	Sub-Theme
The role of family in the medical communication and care process	Information gathering
	Communication mediator
	Intervention in decision-making
	Financial support
	Integrated care
	Adult children as decision-makers
Limitations of family involvement in medical communication	Discrepancies of opinions
	Information concealment by family
	Excluding patient’s opinion

The participants emphasized the crucial role of the family in the health communication process of cancer patients. Families were found to take on a variety of roles in medical communication. First, as *information gatherers*, families actively sought information on behalf of the patients, particularly when the patients were experiencing psychological distress following diagnosis or lacked sufficient health literacy to fully understand their condition. Information gathering occurred not only through interactions with physicians but also through various media sources. Second, families often acted as *communication mediators*. When patients had difficulty understanding physicians during consultations or comprehending the results of medical evaluations, family members accompanied the patient, interpreted and organized the physicians’ explanations, and relayed them to the patient in an understandable manner. Furthermore, families mediated the conflicts that arose when patients mistrusted their physicians or were unable to communicate effectively. In some cases, families persuaded patients to accept treatment even when they were initially reluctant. Third, families provided both financial and emotional support, particularly when the patient was unwilling to pursue treatment because of the anticipated financial burden. In addition to assuming the actual cost of care, families also offered psychological encouragement to ensure that communication with physicians regarding treatment options remained open and constructive. Fourth, families engaged in *integrated care*, extending beyond mediating medical communication to comprehensively supporting patients’ daily life needs, which lay outside the scope of hospital-based treatments. Finally, in the case of elderly patients, adult children often play a more proactive role in medical decision-making. In these instances, the children not only mediated communication but also emerged as primary decision-makers.

#### Information gathering.

Patients with cancer tend to consult their families about whether to visit a university hospital before meeting a doctor, especially after receiving health check-up results or developing symptoms. Adult children often take the initiative in searching for information and scheduling appointments. Additionally, when patients exhibit suspicious symptoms, family members arrange the necessary examinations to demonstrate their involvement in the early stages of cancer detection.

Given the nature of university hospitals in which medical specialties are finely divided, patients must choose an appropriate department for their appointment in advance. Patients lacking medical knowledge or those who find it difficult to search for information often face challenges. Families intervene by collecting information at various levels before medical consultations. This information gathering by families includes self-diagnosis related to the types of cancer correlated with symptoms and the relevant medical departments to visit as well as integrated considerations that encompass post-diagnosis treatment processes, such as who the renowned professors are and what treatment options are available.

I also started looking things up ahead of time, using what I already knew, and searching for a lot of information online. I wanted to figure out how far we should go with the surgery and what the best options are to make sure the cancer doesn’t come back. Since I’m the caregiver, I know that caregivers usually look into things more, so I did my research, and then we asked the professor about any questions we had.

-029, Family (daughter)

I feel like the general public just doesn’t have enough medical knowledge. That really hit me when we were going through my dad’s diagnosis and treatment plan—I was honestly surprised. Like, I remember thinking, *‘Wow, my dad really didn’t know much about this?’* Maybe it’s because I’ve been around hospitals a lot and had more exposure, but still, I was shocked at how little he knew. And he’s actually pretty young! It made me realize just how little insight some people have about medical stuff.

-008, Family (daughter)

In the case of Participant 008, her father was unable to accurately understand the physicians’ statements during their consultation, resulting in failure to recognize his diagnosis of pancreatic cancer. Participant 008 was required to explain the details of the consultations. As a daughter and employee of a university hospital, Participant 008 was involved in every aspect of medical communication, including obtaining information, selecting the hospital, accompanying her father to appointments, interpreting the consultation content, and scheduling subsequent treatments. Without her involvement, her father would have struggled to understand his health condition. Participant 008 illustrates how families participated as communication mediators across all aspects of treatment.

Families not only provided information but also tended to accompany patients to medical appointments. When patients are first confronted with a diagnosis of a serious illness such as cancer, they may struggle emotionally and feel overwhelmed. In such instances, family members assist in scheduling appointments and accompanying the patient, offering emotional support while also helping them understand any details that they may not fully grasp due to mental shock.

I went with my husband every single time, from start to finish, and the reason is that I wasn’t really in the right state of mind. I couldn’t really take in what was being said in the consultation room. So, I asked my husband to pay close attention to what the professor was saying and even to record important conversations on days when we were getting results. I never went alone; he was always there with me.

-025, Patient

When there’s a caregiver, they usually come in together. Most of the time, the caregivers are always there with the patients. It feels like about two-thirds of the time, people come in with their spouses or children.

-037, Doctor

#### Communication mediator.

When patients struggle to comprehend medical information and effectively engage in discussions with healthcare providers and family members acting as caregivers, family members perceive themselves as crucial intermediaries in physician–patient communication.

Family members frequently bridge this communication gap by simplifying and explaining medical information to patients after consultation. Additionally, they may engage in discussions with physicians on behalf of patients during medical appointments to ensure that the necessary information is communicated and understood. Healthcare practitioners recognize that communication during medical appointments is often centered on family members, which is reflected in their interactions during consultations. This is especially true for elderly patients, as medical practitioners tend to prioritize communication with caregivers rather than with patients themselves.

Patients also reach out directly, but from what I’ve seen, usually around their mid-60s to 70s, I tend to contact the caregivers first. Then, the caregivers pass the information along to the patients. I always ask, *‘Who should I contact first?’* and most of the time, patients prefer that I reach out to their kids first.

-012, Nurse

Caregivers often help out and summarize things, especially for elderly patients. This is because many older individuals might not manage self-care very well. When the caregiver is there to summarize how the patient has been doing, it makes things a lot easier for us. For elderly patients, having a caregiver around is much more comfortable and helpful.

-037, Doctor

When miscommunication arises between patients and physicians, family members serve as intermediaries, helping to clarify misunderstandings and reinforce a shared understanding of the treatment plan. The case of Participant 015 demonstrates this:

My father-in-law suddenly asked me, *‘Do you think the doctor is lying to me?’* And I was like, *‘What possible reason would that young professor at a university hospital have to lie to an elderly patient like you? What would they even gain from that?’* I had no idea what exactly he thought he was being lied to about. I didn’t know what to say or how to explain things to him… (omitted)…Then, later, the doctor actually called me. They said my father-in-law wasn’t understanding the situation and asked me to help convince him. It was so exhausting, being stuck in the middle.

-015, Family (daughter-in-law)

So, my mom often didn’t know who to ask about things at first. She would just ask me. I would tell her, *‘I can handle the scheduling later.’* I would step in a bit and say things like, *‘You can ask about this later’* to help her out.

-027, Family (daughter)

The mother of Participant 027 lacked a clear understanding of how to communicate with physicians. As a result, Participant 027 assumed the role of a discerner, identifying which important questions needed to be asked within the limited time, and guiding the patient on which questions were appropriate to raise with the doctors.

For someone going through surgery for the first time, it’s impossible to understand everything perfectly right away. That’s just how it is. I believe it’s also important for caregivers to step in and help patients understand things better. Especially for elderly patients—it just makes sense that they’d need that extra support.

-020, Family (partner)

Participant 020 emphasized the essential role of family members in facilitating the patients’ understanding during medical communication.

#### Intervention in decision-making.

While some patients made treatment decisions independently, our findings indicate that in most cases, decisions were made in consultation with family members or, in some instances, by family members on behalf of the patient. The family’s role became particularly prominent when patients lacked motivation to pursue treatment, as family members often played an important role in persuading them to proceed with medical care.

I think I received a lot of help from my family and those around me who have had similar experiences. The doctor simply explains the options that I can choose. All I could do was ask about the advantages and disadvantages of robotic procedures compared to regular procedures. However, those who have experienced it tend to know more details.

-006, Patient

This influence was particularly evident when a family member had a medical background as patients tended to defer their judgment and decisions. Participant 006’s family member (daughter) worked at a hospital, so participant 006 sought her opinion before making decisions. As caregivers are deeply involved in the patients’ daily lives, their repeated encouragement and persuasion are more effective in promoting adherence to treatment than a physician’s recommendation alone.

I think it’s something that needs to be discussed together. In my case, I tend to rely a lot on my family. For instance, when it came to treatment options and transplants — like the three choices of allogeneic, unrelated donor, and cord blood transplant — I discussed them a lot with my family. Ultimately, we could only go with cord blood, but we had many discussions.

-046, Patient

My grandma was pretty negative about the whole treatment from the start. She kept saying, *‘I’m going to die anyway, so why waste money on this?’* She also didn’t really like taking her medication, so my aunt had to constantly check on her and remind her to take it. She also gave her a lot of emotional support, which I think was really important. I ended up convincing my grandma, and since I work at a hospital, she kind of saw my words as the final answer. She was like, *‘Okay, I’ll do whatever you say.’* So, I think that helped her make a decision in the end.

-024, Family (granddaughter)

When patients did not wish to pursue treatment or rejected a physician’s recommendations, their families played an important role in persuading them to undergo treatment. Participant 006 stated that she consulted her family members when deciding on the surgical method and occasionally sought information from relatives who had experienced cancer. She noted that, nowadays, quite a few people around us have had cancer, but this often remains unknown until they disclose it themselves. When she sought opinions about her condition, she discovered that some relatives had undergone similar experiences. She obtained information from them to aid her medical decision-making. As seen in the case of Participant 024, older patients often felt burdened by the continuation of challenging cancer treatments. However, all family members of the study participants reported that they successfully persuaded them to continue treatment. Through these direct and indirect interventions, family members facilitated patients’ acceptance of treatment, ensuring that financial concerns did not become a barrier to receiving the necessary medical care.

#### Financial support.

In cases where patients were reluctant to undergo treatment owing to financial concerns, family members might play an important role in influencing their decision-making. Some family members directly covered the medical expenses, whereas others reassured patients that the cost would not be an issue, thereby alleviating their financial concerns. The study found cases in which their children discussed and shared costs that were not covered by insurance (Participants 024 and 025). In such cases, the children would often gather or create group chats to discuss financial issues. Importantly, the costs incurred by families ranged from surgery to immediate treatment. Participant 015 stated, *“We [family members] not only covered the surgery fees but also bought functional foods whenever the father-in-law [the patient] wanted them.”* Families also took on expenses related to post-treatment care, such as nursing home fees for rehabilitation, professional caregiver costs, and even health supplements deemed beneficial for cancer patients, thus encompassing the financial responsibility for the entire life of the cancer patient beyond just the realm of treatment.

The costs you mentioned were really important. We discussed whether they were covered by insurance, and if not, who would bear the expenses. After the surgery and discharge, we also talked about who would go to the hospital for care and how to cover those costs. I heard that these discussions happened among the adults involved.

-024, Family (granddaughter)

One of the saddest aspects of providing care is that there are cases where patients end up giving up. This can happen for various reasons, including financial constraints or a lack of someone to provide care. Sometimes, caregivers also don’t make treatment decisions at all, which can be really disheartening.

-044, Doctor

Participant 044 highlighted the absence of a family member or a situation in which the caregiver could not pay the costs as a significant reason for cancer patients to forgo medical care. When families shoulder the financial and caregiving burdens of treatment, a lack of cooperation from family members might result in patients discontinuing treatment regardless of their personal wishes.

#### Integrated care.

In addition to directly participating in medical communications, family members provide emotional support to patients. When patients feel exhausted or consider abandoning the arduous treatment process, emotional encouragement from their families helps sustain their motivation to continue the treatment. Thus, caregivers indirectly influenced decision-making by fostering patient resilience.

After the treatment, since people were going to find out anyway, my acquaintances and family also came to know about it, and sharing those experiences provided great comfort. It made me realize that I’m not living in a world alone.

-003, Patient

At first, we went together—especially when they were getting admitted to the hospital and all that. In the beginning, he was really scared about the immunotherapy, like *really* scared, so I went with him. But then, after actually going through it, we realized it was over in a flash. It felt kind of anticlimactic. So, I told him, *‘Next time, you can come by yourself.’* In a way, that’s also a form of support—helping them feel comfortable enough to go on their own.

-035, Family (partner)

Patients with cancer often experience significant psychological distress upon diagnosis because cancer is commonly perceived as a life-threatening disease [23,24]. As demonstrated in Participant 035’s partner’s response, patients may develop a heightened sense of fear throughout the treatment process owing to the side effects of anticancer therapy, making them reluctant to visit the hospital alone. In such cases, caregivers accompany patients, provide emotional reassurance, and reinforce their commitment to the ongoing treatment. This highlights the indispensable role of family members in supporting patients not only in medical decision-making, but also in their overall psychological well-being throughout the cancer journey.

If the illness I have isn’t something that can be completely cured, then the real question is—can I still live a normal daily life while managing it? Of course, the ultimate goal should be a cure, but if this is something I have to live with for the rest of my life, then the focus shouldn’t just be on the cure itself. It should also be about how I can coexist with this illness and still maintain my everyday life. That’s what really matters.

-014, Patient

One of the most frequently mentioned areas in which patients perceived a critical need for communication with their physicians was detailed sharing and consultation regarding the overall treatment process beyond immediate medical interventions. Although patients underwent regular medical consultations, these interactions often focused primarily on necessary tests and diagnostic results. Consequently, there was a notable lack of guidance on the overall treatment trajectory and the specific roles and responsibilities of patients within that process, along with practical considerations for maintaining the patient’s daily life. Participant 014’s argument that cancer treatment is not just about a cure but about how to coexist with cancer in her daily life demonstrates a different perspective on the cancer treatment.

From the doctor’s perspective, he thinks the improvement is all because of the chemotherapy. But from the family’s point of view, sure, the chemo helps, but we also believe it’s because of all the other things we’re doing—like additional treatments, making lifestyle changes, exercising, and putting in the effort every day.

-009, Family (son)

Participant 009 noted that doctors tended to assess patient improvement based solely on the efficacy of anticancer therapy, whereas patients and their families perceived progress as a result of a combination of factors, including dietary habits, physical activity, and complementary therapies.

I think I unconsciously started to try to understand my husband as much as possible. I didn’t really nag him much. When he was discharged from the hospital, I thought right away that I needed to make sure he ate properly. I decided to switch his diet to natural foods, like really natural. So, I started making tomato soup. You know, like with about 7 or 8 different kinds of veggies in it.

-035, Family (partner)

In this process, integrated care provided by families might be important. Participant 035 demonstrated that families take responsibility for holistic care in areas of daily life that healthcare providers cannot address. They intervene when there is a mismatch between the *treatment* that the physicians aim to provide and the *daily care* that patients require. Dietary management is crucial for cancer patients, and families perform everyday management tasks that complement medical treatment. For instance, Participant 035 reported that following her husband’s cancer diagnosis, she completely revised his diet and deliberately reduced her habitual reminders to minimize his stress.

I don’t think shared decision-making is just something we talk about when someone is first diagnosed or just during initial treatments. We have to keep making ongoing decisions each time there’s follow-up. The family’s involvement in the treatment is just as significant as the patient’s, even though the patient’s opinion is the most important. I believe the family should be fully informed too. It’s all about moving forward together as a family.

-039, Doctor

Healthcare professionals also recognize the significance of families as central figures in integrated care. The medical practitioners who participated in this study acknowledged that while there are both positive and negative impacts on families involved in treatment, they recognize the importance of their role.

#### Adult children as decision-makers.

In the South Korean healthcare context, there are many instances in which family members of patients with cancer are deeply involved in communication, particularly when children are in the caregiver position [25]. Among the study participants, most older patients were accompanied by their children to medical appointments, and the children participated in medical communication. There were also cases in which the children communicated directly with physicians. Interestingly, even when patients with cancer did not disclose their diagnoses to their parents, they indicated that they would intervene more if their parents were diagnosed with cancer. This highlights that despite various family dynamics, communication within the family unit, especially in parent–child relationships, is prevalent in medical contexts.

Cancer patients tend to be older, you know, many are grandparents. A lot of times, their children just can’t accept them not getting treatment. I think there’s still a lot of that Confucian influence around. From what I see, not getting treatment is seen as being unfilial.

-011, Nurse

You know, given their age, elderly parents might not be familiar with the process or able to make rational, logical decisions as easily. So, I think we, as younger folks, would discuss and share the situation with my brother and the rest of the family to make decisions. We’d definitely discuss with our parents, like saying, *‘The situation is this and that, so it might be better to choose this option. What do you think?’* But we’d likely focus on providing them with information that helps them make a more rational decision.

-003, Patient

Within families, the relationships between parents and their children may differ from those between parents and their spouses. One reason why children become deeply involved in their parents’ medical issues, as noted by Participant 011, may be traditional cultural values, in which a child’s involvement in their parents’ treatment is seen as a moral expression of filial piety. However, for older patients, declining physical abilities often make communication with physicians physically challenging and necessitates the involvement of their children. Participant 003, a patient with cancer in their 50s, did not initially inform his parents of his cancer diagnosis. However, he mentioned that if his parents had been diagnosed with cancer he would actively involve himself. This illustrates that in Korean family dynamics, the direction and extent of involvement may vary depending on whether the position within the parent–child relationship is that of the parent or the child.

### Limitations of family involvement in medical communication

In Korean society, while families play a crucial role in medical communication by participating and potentially alleviating communicative inequities, their deep involvement may not always be positive. There are diverse actors within a family, which can lead to differing opinions on who should be responsible for patient care and what treatment should be pursued. If communication within a family is not smooth, it can lead to medical communication issues. When a family has the decision-making authority, disagreements among its members can become barriers to communication.

#### Discrepancies of opinion between family and patients.

Because family members often hold differing opinions, disagreements within the family can hinder effective communication with physicians. Participant 039 noted that when family members fail to reach a consensus or when a relative who has not previously served as the primary caregiver suddenly becomes involved in the parent’s treatment, such situations can disrupt the long-term treatment plan.

Sometimes, people need someone to blame for the situation, and the family can have all sorts of different opinions. Even when the main caregiver is doing a great job, another sibling might come along later and start complaining or blaming them. There are cases where, at the very last stage, someone decides to show up and suggests transferring to a higher-ranked hospital. Like, a son who’s never visited in three years might suddenly call and say he’s booked a spot at XX Hospital.

-039, Doctor

Communicating within the family can be really tough. Everyone has their own opinions and circumstances. Like, some might want to be involved in medical appointments but can’t actually be there. Yet they still have expectations. And then, when the family talks about their opinions, it’s like they just don’t get what I’m going through, and I ended up crying because of it. After that, they said they were sorry.

-015, Family (daughter)

In cases where families persuade patients to proceed with treatment, family members often experience regret and responsibility, leading them to advocate for treatment, even when the patient does not consent. In particular, when the patient is an elderly parent, they may continue treatment at the insistence of their children even if they do not wish to. In such instances, parents tended to follow their children’s opinions. The context of treatment persuasion varies depending on who advocates it (e.g., family members who provide caregiving or those working in the medical field tend to be more persuasive) and the narratives used in the persuasion process. This points to the peculiarities of parent–child relationships in South Korean society.

My grandma was saying she couldn’t stand the side effects of [cancer treatment] anymore. It was a treatment we recommended but seeing her do that made me wonder if it was the right decision. Watching her struggle with the side effects felt really uncomfortable at the time.

-021, Family (granddaughter)

Participant 021 described a case in which the family persuaded a reluctant elderly patient to undergo treatment. However, during chemotherapy, the patient experienced severe itching and skin peeling, necessitating the use of a strong ointment. Observing this, Participant 021 felt a sense of moral burden in proceeding with treatment that the patient did not desire.

Just being in the hospital for a long time doesn’t necessarily mean they’re going to get better. From what I’ve seen, the fatigue among family members just keeps increasing. But the caregivers often find it hard to let go. Even when the chances are super low, they continue with treatments for the patient. Seeing all that makes me think that knowing when to appropriately stop treatment is really important.

-016, Nurse

Participant 016, as a nurse, criticized the reality in healthcare settings, where the wishes of terminally ill patients are often overridden by their families, resulting in prolongation of life against the patient’s will. They emphasized that when a patient is suffering and the likelihood of successful treatment is minimal, the preferences of the patient should take precedence over those of the caregiver.

#### Information concealment by the family.

In the interviews, some family members excluded the patients from communicating. Information was controlled by not sharing details or by asking the physician not to directly mention the treatment results to the patient during appointments. Typically, families withhold information because of concerns about emotional shock to the patient due to the severity of their condition, especially in cases involving terminal or serious illnesses.

Some people don’t even know that it’s a terminal situation from the start. Like, once a caregiver brought their mother in a wheelchair, and I was trying to ask about her condition. So, I asked, *‘What did the gastroenterology doctor say about your condition?’* and the patient didn’t really know. Then I asked, *‘You do know it’s pancreatic cancer, right?’* Just as I was about to mention that it’s terminal, the caregiver crossed their arms in an ‘X’ gesture, which meant the consultation ended up taking even longer.

-033, Doctor

Since I was there, I figured I might as well get checked. Suddenly, they came out and said that although not all the blood results were in yet, the white blood cells, red blood cells, and platelets were already not normal. I went to XX Hospital for a bone marrow test, but even though the family all knew, they didn’t tell me. The doctor didn’t tell me either. They just kept saying to wait, so I thought the results weren’t ready yet. When I found out that they had already told my family but not me, I was really angry at the time.

-046, Patient

Participant 046 expressed dissatisfaction upon discovering that despite being the patient, she was the last to learn about her diagnosis, while the entire family already knew. The discomfort experienced by Participant 046 stemmed from the lack of respect for her right to understand her own body. Cases in which families intentionally withhold medical results illustrate that communication inequity is not always mitigated by family involvement; it can be exacerbated, potentially hindering the patients’ ability to communicate effectively with their physician.

#### Excluding the patient’s opinion.

Instances in which families withheld certain information necessary for effective medical communication and made unilateral decisions without considering the patient’s opinion were identified. This contrasts with cases in which families persuaded the patient, which involved family intervention, but resulted in the patient’s agreeing to treatment, with ample interaction and communication between the family and patient in the process. However, in the identified cases, families either made decisions entirely on behalf of the patient who disagreed with or blocked all the information necessary for the patient to understand the issue, thereby excluding their participation.

It seems like when families struggle to find that balance, patients often end up feeling excluded. This happens a lot with elderly patients, and I’ve seen it with some families. For example, sometimes the son completely takes over the decision-making, leaving the patient out of it entirely. (Interviewer: Even if the patient has decision-making capacity?) Yeah, there are cases like that.

-032, Doctor

People in their 60s and 70s sometimes make their own decisions about surgery, but there are also cases where they come in influenced by their caregivers. So, when they arrive, even if the patient (the mother) wants to say something, the son tells her, *‘Mom, just be quiet.’* And then the son ends up saying to me, *‘Doctor, please schedule the surgery for me*.’ I’m sure she had something she wanted to say.

-044, Doctor

Participant 032 described a situation in which a son with an elderly father did not consider his father’s opinion when making decisions regarding his cancer treatment. Similarly, Participant 044 highlighted a scenario in which a son accompanied his mother, a cancer patient, to a medical appointment and blocked her from attempting to communicate with the doctor about her treatment.

Considering the complex dynamics of Korean kinship relations, the authority of an adult middle-aged son within a family structure can significantly influence how decisions are entirely made by the family. The extent of a family’s influence on communication varies depending on the respective positions of the patient and family members within the family, as well as how these relationships have been shaped over time.

I think family involvement is too strong in our country. Patients suffer because they want to make their own choices, but they feel guilty and worried about what their family thinks. They often wish they could avoid treatment altogether. Regardless, if they have decided to pursue treatment, they should be discussing it with professionals and communicating their thoughts, but family often get in the way of that. I believe the weight of the patient’s choice should increase. There are even cases where some people tell their young patients not to speak up. Parents will say, *‘Don’t say anything*,*’* and send the patient out. There are those who want to talk to me without the patient present.

-011, Nurse

Participant 011 criticized the excessive involvement of families in medical communication in Korean society. Despite the formal recognition of the patient as the principal decision-maker in medical settings, patients’ choices are often overshadowed by familial influences. Instances in which families attempt to exclude patients from consultations and communicate directly with doctors underscore how excessive family involvement can undermine patient autonomy and serve as a direct barrier to effective communication.

## Discussion

This study investigated the role of family members in medical communication involving cancer patients in the South Korean healthcare context. Our findings revealed that families were active participants, not passive observers, in the medical journey, which significantly influenced communication dynamics and patient care. This aligns with and expands upon the existing literature, highlighting the importance of social support networks in healthcare [11] and the concept of health/communicative labor [20], which emphasizes diverse actors in medical communication settings, including the often-unrecognized communicative work performed by nonmedical actors. In their efforts to save the patient, the families’ encounters with numerous medical professionals, and the communicative labor involved, are reflected in their caregiving practices. This highlights the obstacles they face throughout the process [26]. Families gather, interpret, and translate medical information, bridging communication gaps arising from limited consultation times and complex medical terminology [27]. By organizing information, families often act as communication intermediaries, forming coalitions with physicians and facilitating the long-term treatment of patients in various ways [28]. The reliance on families for information mediation highlights a potential systemic issue within the South Korean healthcare system in which time constraints may hinder effective direct communication between physicians and patients.

Families exert considerable influence on treatment decisions and sometimes prioritize familial opinions over patient autonomy [18]. Medical communication does not occur solely during face-to-face interactions with physicians but develops through complex interactions among various actors throughout the entire process, from consultation to post-treatment [29]. Norms within the medical field define the specific types and modes of communication necessary for diagnosis and treatment as valuable, making it challenging to encompass the broader scope of health/communicative labor desired by families. Families play a key role in alleviating the communication burden experienced by patients with cancer during all stages. This integrated caregiving role extends beyond the biomedical realm and encompasses dietary management, lifestyle adjustment, and emotional reassurance. This finding underscores the importance of integrated care models that recognize and value the contributions of families in promoting patient health.

Although family involvement is generally positive, our study identified potential limitations. Disagreements among family members can create communication barriers, and the concealment of information, while often motivated by concern, can undermine the patient’s trust and autonomy [30,31]. This influence raises ethical considerations regarding patient autonomy and the potential for coercion [32]. Disagreements between patients and caregivers frequently arise regarding whether to continue treatment despite side effects, whether to report these side effects to a physician, or opinions regarding hospice care [33]. This study also highlighted points of disagreement concerning treatment decisions, including extreme cases in which families made unilateral decisions without consulting the patients. This study specifically illustrates that in the context of Korean medical culture, family involvement might undermine patient decision-making autonomy. These findings highlight the need for nuanced approaches to family involvement that prioritize patient-centered care and respect individual preferences.

This study is significant in that it provides evidence of the importance of family involvement in developing a Korean model of shared decision-making. In Western countries, although family members are involved in medical decision-making, the final decision tends to prioritize the patient’s opinion [34]. In contrast, in China, Japan, and Korea, patients are more likely to align with their families’ opinions, even when there is disagreement among family members [35]. Japanese patients tend to view medical decision-making as an interdependent sharing of information, whereas American respondents place greater value on patient independence [36]. In the case of cancer patients in China, they place significant importance on their family’s opinions and circumstances when making decisions [37,38]. This suggests that when applying the shared decision-making model to East Asian cultures, it is important to consider the unique medical cultures of these regions. Some studies suggest that the role of the family in medical decision-making is influenced more by individual tendencies within a country than by cultural differences between countries [39,40]. It is important to avoid falling into cultural stereotypes when discussing the differences between Eastern and Western medical cultures [40]. Rather than emphasizing the cultural differences, the significance of this study lies in its empirical demonstration that within Korea’s unique medical context, families continue to play an important role in health communication. The existing shared decision-making model based on Western European healthcare systems consists of three stages: team, option, and decision talk [41]. This study demonstrated that, in the Korean cultural context, families are involved in various verbal and nonverbal forms at every stage of medical communication. This suggests that the Korean model of shared decision-making should include stages that account for family involvement.

The limitations of this study include its qualitative design and relatively small sample size, which limit the generalizability of the findings to a broader Korean population. Although this study included cancer patients at various stages of the disease, the interview questions were not tailored to each stage. Moreover, the sample sizes for each stage were not sufficiently balanced to enable a clear distinction between stage-specific experiences, which is a limitation of this study. This study also had methodological limitations, as patients, nurses, and family members were mostly recruited from two university hospitals. Consequently, these findings may not fully capture the geographical diversity or heterogeneity of cancer treatment facilities. Future research should employ larger mixed-methods studies to validate these findings and explore the cultural context of family decision-making in greater depth. For instance, comparing the general financial support provided by family members with the actual frequency of their accompaniment to medical appointments, as well as analyzing their narratives, would offer meaningful academic insights. Finally, the long-term impacts of these communication patterns and family roles on patient well-being and treatment outcomes warrant further investigation.
